# "The Hospital" Nursing Mirror

**Published:** 1897-09-04

**Authors:** 


### "The Hospital" Nursing Mirror.

Being the Nursing Section of "The Hospital."

[Contributions for this Section of "The Hospital" should be addressed to the Editor, The Hospital, 28 & 29, Southampton Street, Strand, London, W.C., and should have the word "Nursing" plainly written in left-hand top corner of the envelope.]

### News from the Nursing World.

### A GIFT TO THE QUEEN.

The women of England who have married Germans, and some gentlemen associated with them, have presented the Queen with a sum of money to be added to the Jubilee Nurses' Fund, an album with the names of the subscribers, and a diamond and emerald bracelet as a personal souvenir in honour of this triumph year of Her Majesty's reign. Mr. Arthur Bigge replied to the Princess Henry of Pless, through whom the gift was made, expressing Her Majesty's sincere thanks at the "generous undertaking," and her pleasure that the Queen's Nurses should reap the benefit.

### A FIRST AID DEMONSTRATION AT CARISBROOK CASTLE.

H.R.H. Princess Victoria of Schleswig-Holstein, attended by Baroness von Zu Egloffstein and Captain Martin, was present at the annual demonstration of St. John Ambulance Association on the Bowling Green, Carisbrook Castle, and gave those present of the successful candidates their awards. The Princess arrived about half-past five, and was presented with a bouquet by Miss Evelegh. The members of the women's classes began the demonstration by showing their skill in bandaging. The prize-giving followed, after which the programme was gone through, finishing with a march-past. Between four and five hundred persons were present, who took the greatest interest in the displays, and applauded the most expert. All the local secretaries and lecturers were presented to the Princess. The Ventnor Volunteer Band gave the music.

### JUVENILE BEGGARS.

Whilst the question of how best to clear the streets of juvenile beggars occupies the attention of social reformers, short-sighted philanthropists send their children out into the thoroughfares to beg for their favourite charities, and for the sake of a few pounds subject them to a humiliation that has a bad moral effect. Here is a case in point. The Hucknall Torkard Nurses' Home is undoubtedly an institution deserving public support, and popular with the people, but does that make it right to dress up a number of schoolgirls in nurses' costume, and send them to perambulate the streets to the music of gratuitously-played temperance bands? Is not the £8 thus collected dearly bought? And what do nurses say to this use being made of their uniform?

### AN IRISH POOR-LAW SCANDAL.

Much is said and written of nurses being overworked, but nothing carries a point home to the imagination like a well-authenticated fact, and a fact brought forward for some other purpose is often more telling than one cited for the occasion. Whilst the Lurgan Board of Guardians were discussing whether it were necessary to appoint one night nurse or two, or whether the Local Government Board would allow them to have two probationers instead of a trained nurse, the amount of work actually done by the retiring night nurse transpired. There are 180 patients in the infirmaries, of which number 20 to 30 frequently require strict attention, and once the nurse had had to attend two confinements and two deaths on the same night, her only available assistant being a pauper bribed by extra rations to sit up. Is it credible that there are men who, but for the fear of the Local Government Board, would put such a load on the shoulders of a couple of inexperienced girls, and be guilty of such injury to the sick? The medical officer, whose opinion ought to be decisive in a case like this, asked for two night nurses, but the Guardians in their superior wisdom will only grant one, and the motion that a probationer assistant be appointed to help her was lost by a large majority.

### CITY OF LONDON UNION INFIRMARY.

The scheme for the reorganisation of the medical and nursing staffs of the City of London Infirmary is now being carried out. The plans drawn up by Mr. Pridmore for structural alterations have been forwarded to the Local Government Board, and tenders have been asked for from several firms. The cost is estimated at £1,250.

### RECONSTRUCTION OF THURLOW NURSING ASSOCIATION.

Some twelve years ago a fund was collected as a memorial to the late Chancellor Thurlow and devoted to the sick nursing of the parish of Malpas. Since then a nurse has been steadily at work; but as the district covers about forty-five square miles, and the demand for the nurse's services has increased, she could not do all there was to do, so the committee have had to find some way out of the difficulty. At a public meeting called for this purpose a resolution was carried unanimously dividing Malpas and the parishes of Bickerton, Tushingham, Cholmondeley, and Whitewell into two districts, each district to have its own nurse, and each empowered to draw upon the Thurlow fund in proportion to its population. By this scheme Malpas loses about £40 per annum (£18 from the fund and the rest from outlying subscribers). Up to the present time the nurse has cost from £65 to £75 per annum. As the income has been £65 only, the deficiency has been met out of a reserve fund accumulated in more prosperous years. It is to be hoped that the committee, which remains unchanged, will manage to balance income and expenditure more fairly. The Rector announced at the end of the meeting that he could give £15 a year from charities under his control towards covering the loss. Another £30 per annum would, therefore, be enough for all purposes.

### NURSES' HOME, BRADFORD GENERAL INFIRMARY.

The Duke of Devonshire laid the foundation-stone of the Infirmary Nursing Home on August 30th. The stone was laid with Masonic honours. The members of the different lodges assembled at the Masonic Hall, Rawson Square, and marched in procession to the infirmary. The new home will cost £10,000, and has accommodation for 50 nurses.

### AS OTHERS SEE THE DISTRICT NURSE.

Actors on the stage proverbially see least of the play. By the same rule, nurses as a class see and know least of the prejudice against them amongst the poor. Not long since a commercial co-operative society was asked for a contribution towards the funds of a newly-started district nursing association. The request was refused on the grounds that "the directors did not consider themselves justified in applying the shareholders' money for such a purpose," and "because they did not approve of the manner in which the association had been sprung upon them." A letter from a shareholder to the local journal on the subject explained matters more clearly. "We don't want," he wrote, "nurses and those who pay them to come spying round our homes, learning our secrets, and taking note of our little tiffs. When anyone is sick, the proper nurses are that person's relations." Nurses of the right kind will dispel this prejudice; nevertheless, it exists, and those training for district work must be prepared to face it.

### CUMBERLAND COUNTY NURSING ASSOCIATION.

The Executive Committee of the new County Nursing Association has been appointed. Lady Mabel Howard is acting secretary, and Mrs. Irwin acting treasurer. The Lord Lieutenant headed the movement, which is to be an expression of rejoicing at the Queen's long reign. It will train and supply nurses for the poor, and will be supported by private subscriptions, though the various county councils and boards of guardians will be asked to help. The donations up to the present amount to £283, the annual subscriptions to £50 per annum.

### DOUGLAS NURSING ASSOCIATION.

The Bishop of the diocese preached at the open-air service held on Douglas Head, and made a stirring appeal on behalf of the nursing association. Notwithstanding the rain a large congregation assembled, and many remained until the end. The collection amounted to £6. The fund is £50 in debt, although there is only one nurse, and she is well employed. The population numbers 20,000, and nearly double that during the summer holidays, a fact that proves how many there must be who never give to charity. Twenty-thousand shillings is £1,000, and yet the nursing fund is in want.

### CORNWALL NURSING ASSOCIATION.

On August 20th the Executive Committee of the Cornwall Nursing Association held a meeting, at which Miss Peter, of Q.V.J.I., was present. The necessary money has been received, although no appeal has been made for it. About £1,159 has been promised as donations, and £35 per annum in subscriptions. The actual amount paid reaches £560. The outlay, so far, has been £100 voted to the Queen's Nurses' Fund, and current expenses. Arrangements were made with Miss Peter to appoint Miss Michie first superintendent, to begin work on November 1st, provided she would undertake the instruction of classes. Miss Peter also promised to engage nurses if possible for the same date, pending the training of suitable women. A resolution was passed asking Miss Peter to appoint a lady competent to give technical instruction in nursing for the county during the month of October; also that a request for a contribution to her salary and expenses be made to the Technical Instruction Committee of the County Council. The superintendent's salary is to be £50 per annum, board, lodging, and travelling expenses; her headquarters will be with the St. Austell Association, and her work will be at first to look after the village nurses, as the Queen's nurses are under the inspection of the central institution. Efforts are to be made to instruct the people beforehand by means of leaflets on the work of the nurses and the advantages offered by it, in order to break down prejudice, and ensure the newcomers a welcome.

### THE INDIAN NURSING SERVICE.

Amongst the many clouds of trouble now shrouding India comes the grievances of the nursing service. The reason is not far to seek. Famine, plague, and war put a strain on this service that makes all weak places plain. The most serious wrong alike to the army and those nursing it is that the service is understaffed. With an exhausting climate, sudden emergencies, and unforeseen contingencies, it is worse than folly to keep nurses overworked. Yet at the present moment it not infrequently happens that one nurse is doing the work of two. Another wrong to the nurse is the fact that she is not well paid, no extra allowance is made in expensive districts, as in Burmah, and she is taxed as a civilian, although the service is an integral part of the Military Medical Service. Nor are deficiencies in present pay made up by future pensions; in fact, home work offers better prospects in this respect.

### SHORT ITEMS.

A trained nurse is to be appointed to take charge of the night work in Athy Union, Dublin, The Sisters of Mercy are on duty until ten p.m. under the present arrangement, and untrained paupers attend to the night nursing.—Clara A. Storer, a nurse in the employ of the Oldham Nursing Association, was charged in the Oldham Police-court with a number of petty larcenies. She was convicted and sentenced to three months' hard labour. The prisoner fainted upon hearing her sentence, and afterward struggled violently, screaming with rage until she was shut in her cell.—The Vicar of Ilford, Essex, asks for nursing requisites, such as mackintoshes, air cushions, &c., for the use of the parish nurse. These are expensive, and a lending store would be a boon to the poor.—Castleford District Nursing Association, unless speedy and substantial financial aid be forthcoming, must abandon its work. The work is appreciated, and there being no hospital or dispensary much needed.—Nurse Compton, of the Newport District Nursing Society, continues popular with her patients and appreciated by her committee. During last year she has nursed 89 persons—slightly fewer than the year before—but she has paid more visits. The total income of the society was £89, and there is a balance of £16 left in hand.—An assistant nurse is to be employed when occasion requires at Woburn Workhouse Infirmary. Nurse Jones is returning to her work after having been ill from overstrain. A man named Bodsworth committed suicide as the nurse was not able to attend to her duties by night and day.—A nurse has been appointed to nurse the sick poor at Tayport, N.B., in commemoration of the Queen's year.—Two nurses on the staff of the Swansea Hospital have contracted typhoid from a patient at Kilbrough.—Mr. Lacey, the donor of the Victoria Nursing Home, Thame, Aylesbury, laid the date-stone in the middle of August. The building is nearly complete, and it is expected to be ready for occupation by October.—The fund for endowing the Victoria Sick Poor Nursing Association, Huddersfield, now reaches £2,900, and the subscription roll £45 per annum. It is hoped that the nurses will be at work before the winter sets in.

### Lectures to Surgical Nurses.

By H. A. LATIMER, M.D. (Dunelm), M.R.C.S. of Eng., L.S.A. of London, Consulting Surgeon, Swansea Hospital; President of the Swansea Medical Society; Lecturer and Examiner of the St. John Ambulance Association, &c.

### XII.—METHOD OF TAKING TEMPERATURES—WHERE TO PLACE THE THERMOMETER, AND INFORMATION OBTAINED FROM THE USE OF THAT INSTRUMENT—MODES OF ONSET, CONTINUANCE, AND DECLINE OF FEVERS.

A GREAT deal depends upon the way in which you use the thermometer. Before using it be careful to shake the mercury well below the normal point, for not only are you on the look out for rises above what is right, but you should also be prepared for falls below the same degree. Now you have a choice of places in which to put it—under the tongue, under the armpit, in the groin, in the vagina, or rectum. There are conditions of unconsciousness or restlessness in which you cannot safely place it under the tongue, and in young children it is quite impossible to do so, as they will not cover the bulb of the instrument with the tongue, and so they render the observation worthless, for unless the bulb lies covered in this way it is worse than useless to place any faith in what is recorded. I do not believe in temperatures taken at the armpit, for I have often found them to be erroneous records, even when care has been exercised to see that the bulb has been well placed, and that the arm has been closely applied to the side. I gave up taking temperatures there some years ago, after having proved the fallacy of it several times. In children you can get a very fair observation by carefully packing the bulb and some of the instrument itself in the crease of the groin, and then, when one leg is lifted over and placed on its fellow, the part is practically converted into a closed cavity, with a proportionally good result. But you cannot well do this in adults, and you gain nothing by it if you do, for, when they are unconscious, you can be surer of a true record by placing the instrument in the rectum than in any other situation. You may have to take comparative temperatures by placing one thermometer in the mouth, and another in the bowel or vagina, for when fever has its origin in local inflammation we often find wide differences in the temperatures recorded in the two places, and so obtain valuable information as to its seat. Thus, in a case of cancer occurring within the abdomen, which I was attending the other day, the temperature under the tongue was generally normal, while the rectal record was over 100 degrees; and you may have a low temperature in the mouth and a high temperature in the rectum, as in another case I saw recently, when, on being called to a young man who was suffering from the violent inflammation which accompanies the gripping of one portion of the intestine by another, I found the record to read 96 degrees in the former situation and 103 degrees in the latter. It is needless to say that such disproportions indicated a fatal issue to the case.

Other valuable indications are afforded by the systematic use of the thermometer. It heralds the approach of new symptoms and of change of condition in the patient, and often marks definitely the advent of some new complication. Therefore be most careful at all times to make sure of your use of the instrument. If you have the least doubt as to the record being a correct one, shake down the index of the instrument and replace it for a second time. The ordinary clinical thermometer will require five minutes for its action, and it is well to leave the half-minute instruments in place for at least one minute. I have known apparently slight causes affect the record. Thus, not long ago I took the temperature under the tongue with a half-minute thermometer, and found it to be subnormal, when it was obvious that there should have been a higher degree of heat than was normal. Someone then remembered that the patient had been sucking ice just before I had put the thermometer under his tongue, and, on again testing him a little later on, I found the higher temperature which I had expected to have discovered in the first instance. Sometimes patients will deliberately trick you when they want to gain an end. They want to be allowed a richer diet than the one they are taking, or desire to be allowed to be out of bed, and, having noticed that they are forbidden these indulgences in consequence of the prevalence of feverish temperatures in their cases, they deliberately set to work to falsify your records. Thus I knew of one young man who was slowly recovering from an attack of enteric fever. He had been told that the enlargement of his diet depended upon thermometric records obtained by placing the bulb of the instrument under his tongue, and he managed to give favourable ones by displacing the bulb from that situation and holding it in the cavity of the mouth. Little tricks of this sort can be easily overcome by placing the instrument carefully in the chosen spot, and by making sure that it is retained there when it has been placed. On the other hand, invalids are apt to be kept in an anxious state by constant thought about these "takings" of the thermometer, and it is often well to keep the charts, which are a record of them, out of sight so that they may not know what they tell. I have known them in our hospital carefully inspect the charts, and be in a state of great depression from what they have seen there; and it is partly for this reason that the papers are now enclosed in cases and are so removed from ready access and observation.

Disease, as noted by the thermometer, is divided into periods of onset, continuance, and decline, and the record of these different stages is most significant and tell-tale when method is employed in its use. It may commence abruptly by a series of chills, or by one or more severe shivering attacks, in which, although the sufferer feels cold, his body heat has considerably risen; such shivers are called "rigors," and they often betoken a grave condition of affairs. When a rigor occurs the patient is seized abruptly with a general sensation of coldness; he shivers so violently that the very bed on which he lies shakes, his teeth chatter, and his skin is cold and blue in colour. Yet, on taking his temperature, you will find it to be high. The fact is, that owing to an irritant acting through the nervous system on the small blood-vessels of the skin, the latter become contracted in rigors, and the blood is driven out of them to seek the deeper-seated vessels in the interior of the body, and so we have a cold surface to deal with. In other cases the temperature will mount by steady increases from day to day, and then the onset of the disease which we are watching is said to be gradual. During the continuance of the fever, temperatures may be steady, or there may be abrupt rises and falls of the heat waves; and the case may end by a sudden fall from a height to the normal point, or below it, or the patient may gradually have less and less feverishness. The first mode of termination is called "end by crisis," the second "end by lysis." There may be variations of these endings, as by irregularity in the decline of the disease, the temperature falling steadily from day to day, but these are the two great types of return to health from the feverish state, and it is well that you should fix them in your mind. The critical fall of the temperature is often accompanied by some new symptom, such as an attack of diarrhœa, a profuse sweating, a copious flow of urine, or a prolonged sleep; and, if the condition has been rightly appreciated, the medical attendant will decline to interfere with any one of these conditions, as he will recognise them as being Nature's modes of terminating disease. By knowing that these phenomena accompanying critical terminations of the feverish state are natural ones you will, yourselves, cease to be anxious about them, and will be enabled to allay the anxieties of friends of your patients when they arise.

Leaving the subject of heat in fever, we will now pass to another set of disturbances which often arise in that condition. I allude to disorders of the brain—the organ of thought, and the centre of the intellect. When we are in a state of mental health we exercise a control over our thoughts, for we have a "healthy mind in a healthy body," but as soon as we become markedly ill by any process which is accompanied by fever this control over the thoughts and imagination is disturbed, and the mental faculties run riot. Thus it happens that headache, confusion of thoughts, and "light-headedness," or delirium are accompaniments of fever, any one of these conditions being apt to arise during its prevalence. Delirium is shown by the patient talking, singing, or shouting, and the disturbance of the mind which causes it may lead to violent movements and actions, as well as to these excited acts of speech. To understand why it occurs you must be made aware of the fact that although the brain usually receives impressions from without through the channels of feeling, sight, hearing, taste, and smell ("the five gateways of knowledge"), it is capable of originating these special sensations from within, and that unless certain "centres" for these functions existing within that organ are in a healthy condition matters connected with them will all go wrong. Besides these centres of special senses, the brain also possesses intellectual centres, in which various thoughts originate, and memories of past occurrences are stored up.

### Some Radical Defects of the Indian Nursing Service.

In early autumn the Indian trooping season begins. Each year sees the departure from England of new recruits for the Indian Nursing Service, and it is in the hope of benefiting these new members of the service that this paper is written.

Too many nurses rush into the service ignorant of what is before them, carried away by the prospect of what is undoubtedly a good, sure income, and in some cases expecting a social rise, for which they are totally unfitted by their previous education and early training. Again, many well-trained, well-educated women, "ladies" in every sense of that much abused word, come out to India hoping for lighter work, longer hours off duty, and greater freedom than they can ever obtain in a large London hospital. There is yet a third class, perhaps even more deserving of sympathy than the above-mentioned, *i.e.*, those nurses who come out full of professional ardour, thirsting for "good cases" of the many and varied forms of tropical disease, conscious of their power to administer a ward or an hospital, and anxious to add to their knowledge by every possible means.

Now to all those different types of the modern nurse, I would say most emphatically, "Avoid the Indian Nursing Service, for it will neither satisfy your ideals nor fulfil your hopes."

Those nurses who seek merely to "better themselves" socially (laudable though this ambition may be from their own point of view), need not be specially considered. Those who hope for lighter work, &c., will indeed be grievously disappointed. Although none of the scrubbing and cleaning, so well known in one's probationer days, falls to the nursing sister's lot, the intense strain of overseeing the worse than imperfect work of native servants is far greater and far more exhausting in the trying climate than the hardest day's scrubbing ever exacted from the most overworked probationer. Then often, all too often, a large garrison is left with half its proper number of sisters. A rush of work comes, enterics flock in, one sister toils all day and every day, the other is on duty all night and every night, disturbed in her day sleeping hours by native servants, tom-toms, military bands, and all the ceaseless noises which make life hideous in an Indian cantonment. Exhausted in mind and body by many repetitions of such events, both sisters fervently wish themselves back in the comfortable nurses' home of their Alma Mater, and curse the day that saw their embarkation in a trooper.

But harder to bear than these material evils is that sense of being cut off from all intellectual life which besets the nurse who takes a keen interest in the theoretical side of her work. Should it be her happy lot to work under a medical officer who, full of talent himself, can appreciate and encourage the efforts of his subordinates, all will be well. But such a man is indeed a *rara avis* in that happy hunting-ground of impecunious Irishmen—the Army Medical Service. The average medical officer, when he is not absorbed in racing and polo, cares little for theory and less for science. He prefers to rub along in the good old fashion, doling out stereotyped doses of calomel and quinine; content to have his orders carried out in the happy-go-lucky method peculiar to the Eurasian apothecary (who, by the way, has now blossomed into an "assistant surgeon," but his habits remain unchanged). Such a medical officer strongly objects to a highly-trained nurse. She sees his failures and omissions too distinctly, and however deferential and respectful her outward manner, he feels that a critical spirit is lurking underneath, and his *amour propre*, if it exists at all, is too often wounded to allow of peaceful harmonious work.

Perhaps in despair of finding scope for her talents in hospital, this sister turns her attention to society, hoping to find a congenial atmosphere in the company of cultured men and educated women. In this she may be exceptionally fortunate and successful. But she is much more likely to meet the man whose conversation is composed of equal parts of regimental "shop" and station gossip; and the mem sahib, who discourses amiably of the baby's teething (with special references to eye teeth), or descants on the shocking misdeeds of the dhoby.

Or, again, the sister's lot may be cast in some station where her undesirable predecessors have brought the nursing service into social discredit. She will then find herself looked upon with coolness and contempt. Some such remarks as these will come to her unwilling ears, with the emphasis as it is here given. A sick officer is about to be brought to hospital—"O *poor* Mr. So-and-so. *Must* he go to hospital? Can *no*body save him from these abominable women?" And the sensitive, highly-strung woman shrinks into her shell, counting up the weary days and months that must elapse before she can escape from such an atmosphere.

But having tried to sketch the type of nurse who ought not to come to India, let us now consider the nurse who will serve with equal benefit to the service and to herself. A sensible, practical well-trained, well-educated young woman, with iron health and no "nerves," who is anxious to see the world and enlarge her experiences, may do much worse than spend five years in the Indian Nursing Service. She must be prepared for very hard work, too often amid uncongenial surroundings and in a trying climate. Above all things she should possess a keen sense of humour, to carry her through her many battles with native subordinates, and the many rubs and petty trials of Indian daily life. Let her be prepared to "give and take"; let her not expect a bed of roses; let her watch her health most carefully, and she may reasonably expect to return home none the worse for her Indian experiences, with, we will trust, a few hundred rupees in hand as the result of her hard toil.

In conclusion, I would briefly draw attention to the principal defects of the service which the new sister will encounter:—(1) Its overtasked condition, particularly in the Madras Presidency, where the number of sisters allotted to each station is not maintained. (2) Absence of extra allowance for sisters serving in Burma. (3) Payment of income-tax as a civilian, although it is stated in regulations that the Indian Nursing Service "forms an integral part of the Military Medical Service." (4) Absence of pension or pension fund. But, with all its faults the Indian Nursing Service has done good work, and could do much more under improved conditions of service.

### Post-Graduate Clinics for Nurses.

By a Trained Nurse.

### XXVIII.—THE COOKING AND SERVING OF SICK-DIET.

Even though the diet may be limited to three or four possibilities, a clever nurse may ring a great many changes by cunning combinations, while different flavourings will cause the same dish to appear in many fresh disguises. So often one hears a nurse say of a patient, "You see he is only on milk diet, so, of course, he tires of no change, and turns quite sick at the sight of the everlasting milk."

Now, if there is any one diet which admits of infinite variety and most delightful capacity for change of flavour it is a milk diet, as I shall presently proceed to show.

Nurses should be as familiar with the cooking of a patient's food as they are with the taking of his temperature. The only way to successfully learn sick-cookery is to prepare the same dish over and over again.

Some nurses say, "I once made some beef-tea, and I have the recipe for quantities, &c., in my note-book, so I can easily look it up." Now, it is certain patients would lose confidence in their physician were he to say, "Oh yes, all that is wanted is a tonic. I will look up the right quantities of quinine and iron in the Pharmacopœia and send a prescription later." In the same way I should lose all confidence in the fitness and capacity of a private nurse were she unable to repeat without hesitation the formula for beef-tea, the exact method of preparing peptonised milk, or the best recipe for making chicken broth.

Every nurse who elects to do private nursing should be familiar with every detail of diet, and should have at her finger ends the minutiæ of the quantities necessary and the time required to compound every dish in the "Diet Pharmacopœia of the Invalid."

A glorious era for the invalid will be inaugurated on that day when an English training school enacts that no nurse shall be considered to have earned her certificate until she has herself prepared from start to finish, as part of her qualifying examination, at least a dozen invalid dishes selected at the discretion of her examiners, personally tasted, passed, and approved by them.

Then, indeed, will our death-rate diminish and convalescence be quickly established in a large percentage of cases where now the sick person languishes and lingers in picking up strength, all for the need of judicious and trained "kitchen nursing."

So far as food is concerned, the invalid of ten years back was perhaps in some ways better off than is the sick person of to-day. For ten years ago the average woman took far more interest in her household cuisine than is the case at present. And she was probably old-fashioned enough to retain a knowledge of the concocting of delicious chicken broths and veal tea. Chicken broth is a sick-room dainty fast going out of fashion. Yet it would be difficult to invent any more delicious dish to set before a sick person. And veal-tea, the most delicate and delightful drink, of which one retains just a memory, is banished apparently irrevocably from the sick-room. And in most households invalid dishes are left to "cook." The modern mother of the house confesses unblushingly that "she does not understand these things," though she has an infinite and subtle knowledge of bicycle gearing and the intricacies of golf clubs. And, worse than all, the trained, the certificated hospital nurse has been turned adrift from her training school without ever encountering a piece of uncooked meat wherewithal beef tea is made, and absolutely ignorant of the mechanism whereby the crude material of a chicken is evolved into stimulating, nourishing, and altogether delightful broth. Again, her training school sends her forth, equipped by virtue of her certificate to serve food to the sick. But in that same school the only apprenticeship she has gone through in the art of making food attractive to the invalid consists of a somewhat haphazard method of depositing rough mugs of milk or unscientific beef-tea on bare lockers, the mug wet on the bottom with the fluid it contains and leaving a sloppy outline of its form when removed.

There is no attempt in the average hospital to reach the barest standard of daintiness in the serving of food. I know only two hospitals in London where the diet in the public wards is served with real refinement and consideration for the niceties of life. In most others the way in which the food is served is the great blot in the hospital system as the want of systematic teaching on the important question of invalid cooking and attractive serving of food is the great blot on our system of nurse-training. It is perfectly certain, unless the nurse has had exceptional opportunities of learning in her own home the routine of nicely set-out trays of food, that the rough-and-ready system of diet-serving prevalent in hospitals will offer a very ineffectual training ground for work in the private sick room. And this accounts for the complaints so often uttered by private patients, that their trained nurses do not know how to make invalid food tempting and attractive. In some individual cases the nurse will know instinctively how to set forth food in the most palatable and prettiest form. But if so it is in spite of, not because of, the teaching on this point which her training school afforded.

In some training schools it is the custom at the end of the first year to test the skill of the probationers in the padding of splints, the application of bandages and the artistic completeness of their methods of cutting dressings and making absorbent sponges. But I have not heard of a hospital which holds a similar exhibition of the probationer's power in concocting dietetic delights for her patients. One has seen many a probationer of a year's standing quite unable to prepare a really satisfactory piece of toast, and it would be an excellent plan to hold object lessons in these simple matters. In an American training school I have seen a probationer condemned—because she was so inefficient in this direction—to make all the toast needed in the diet-kitchen for a whole week. Each piece turned out was inspected by the head of the diet-kitchen, criticised, and, if necessary, rejected. In the same diet-kitchen many varieties of crockery were kept in different shades and shapes, so that the kitchen pupils might have practice in the art of arranging the most artistic combinations of china for the patients' trays. And I would like to call attention to the fact that this diet-kitchen was not the "fad" of a wealthy hospital receiving paying patients. For it was in a charity hospital, corresponding to an English workhouse infirmary. It seems to me in the training of nurses it would be so easy to establish invalid tray-setting competitions, as a variation on splint-padding, and there is little doubt that nurses would hail such an innovation with delight, as affording them most valuable training without, at the same time, adding to "head-work," and mere "book-cramming."

### Wants and Workers.

[The attention of correspondents is directed to the fact that "Helps in Sickness and to Health" (Scientific Press, 28 & 29, Southampton Street, Strand, London, W.C.) will enable them promptly to find the most suitable accommodation for difficult or special cases.]

Nurse Webster would be glad of a bed rest and books for reading to the sick and well for use in her district.—Compton, Guildford, Surrey.

### Jubilee Keeping in Vancouver.

By a Correspondent.

Being the farthest western point of her Majesty's dominions, it may interest The Hospital reader to know how we kept the Jubilee. On Sunday, the 20th, the Sons of England organised a procession in which all the various societies joined. All marched to the Vancouver Hotel Grounds. Places had been assigned, and then came the Rector of St. James's (Mr. Fiennes-Clinton) and the surpliced choir, and took a position upon a platform prettily draped and arranged with flags and wreaths, and then we had a grand open-air service, the City Band supplying the music. Such a gathering has never before been seen in our city. The mail steamer (one of the three "Empresses" of the Canadian and Pacific line), fired a signal rocket, and simultaneously the Union Jack was run up and the National Anthem sung by all present. This was by our time quarter-past four p.m., so, allowing for the difference in time, we were celebrating with England and at the actual moment. A collection was taken after the service in aid of a new ward being given by the Sons of England to St. Luke's Home. Then, on the 31st, all the nurses of Vancouver assembled in the home to receive the guests for a garden party we were giving. We had the grounds prettily illuminated with gas devices and electric lights placed in the trees. The City Band gave us their services. All our friends sent cakes and strawberries, so we were anticipating a very crowded and enjoyable evening. Alas! at eight p.m. rain came and poured down. The band was here and had played one selection, bringing our nearest neighbours over; fortunately we had cleared out the large ward, fearing it might be damp and some would prefer viewing the scene from the windows; so we all adjourned there. I found an empty room for the band. Our patients were quite convalescent enough not to mind, so we had a small but merry party indoors, about 100 guests arriving. We had expected, however, as many more, as the medical men had promised to be present, for it was not only our Jubilee entertainment but our first nurses' party, as all other institutions and private nurses joined in. Miss Macfie, the matron of the City Hospital, helped me to receive at the garden entrance. We sang the National Anthem at eleven o'clock, and so ended our first entertainment. We all hope, however, not our last Vancouver nurses' party. The several uniforms looked very pretty. We were almost equally matched in nationality, half of us English and half Canadian. The Sons of England are making great efforts to furnish their ward with four beds to be devoted to Englishmen, who can be received at a fixed rate of 5 dols. each per week and enjoy all home comforts and nursing. "The Daughters of England" are doing likewise with one bed for women, so they have given a private room. The ward is to be dedicated and called "Victoria." Her Majesty has approved and also sent her portrait and autograph, which arrived last week, so we are to have the ward opened and the picture hung with great rejoicings when the date is fixed. We are so proud and happy over it all. It is rather a coincidence that the first nursing institution out in the West should be so honoured. And I am the first nurse who came to Vancouver, so I have seen all around me grow up. And now to think our little home and ward is so favoured! We wanted to open the package badly when we saw the Royal seal, but have had to wait till the "Sons of England" saw it, and now it is to be framed, and also the letter from Lord Edward Pelham Clinton, and then I shall be able to write and tell you about the ceremony of opening the ward and presenting the picture. Our Jubilee has been great, and I hope will be lasting and benefit our Englishmen out here. The city of Vancouver set apart July 1st and 2nd for celebration, and, alas, again it is raining. The flag ship, H.M.S. *Imperieuse*, with two smaller, is in port, also two mail steamers, Australian and China, and the town is *en fête*. The weather has cleared enough to begin a cricket match, and I can hear the troops out and bands playing. To-night we are having a high fancy fair in the hotel grounds in aid of the English churches here, so we do hope for fair weather. You will see by my letter we are very loyal here. We have also been very busy collecting for and advocating the Canadian Victorian Order of Nurses, and which I hope in the near future will be a settled order. Like all new things, though, it takes time to teach a new idea, but once established I think it will go ahead.

### Minor Appointments.

Eastern Hospital, The Grove, Homerton.—On August 21st the following Charge Nurses were appointed at the above hospital: Miss Clara Smith, trained for three years at the Royal Berkshire Hospital, Reading, and afterwards charge nurse at the National Hospital, Ventnor, and the Royal Sea-Bathing Infirmary, Margate. She has been for the last three years on the private staff of the General Hospital, Cheltenham. Miss Georgina Trotter, trained for three years at the Sunderland Union Infirmary, and afterwards charge nurse at the Gore Farm Hospital. Miss Eliza Anne Hitchings, trained for three years at the Birmingham Workhouse Infirmary, and afterwards employed for three years district nursing at Dolgelly. Miss Margaret Carr, trained for three years at Royal Infirmary, Halifax, and afterwards charge nurse there for two years.

Great Yarmouth.—Miss Frances Elizabeth Glover was on July 30th appointed Charge Nurse of the Union Male Infirmary. She was trained at St. George's Infirmary, Fulham Road, West Brompton, and has been nurse at Woolwich Union and on the private staff of Hampstead Hospital.

Ecclesall Bierlow.—Miss Ellen Mace has been appointed Charge Nurse of the Union Infirmary. She was trained at Brownlow Hill Infirmary, Liverpool, and has been charge nurse at South Shields Union Infirmary and at Ormskirk Union Infirmary.

General Hospital, Birmingham.—Miss Harriett A. Marriott has been appointed Assistant Matron (not matron as stated last week) of this hospital. Miss Stevenson is the matron.

Shoreditch.—Miss Jardine, who has been appointed Superintendent Nurse of the above infirmary, was five years in the Indian Nursing Service, three years as nursing sister, and two as deputy superintendent.

Teignmouth District Nursing Association. — Miss Roberts, of Manchester, has been appointed Nurse of Teignmouth District Nursing Association, in place of Miss Ravenscroft, resigned. She began work on September 1st.

### Where to Go.

Midwife's Institute and Trained Nurses' Club.—On Thursday September 2nd, at three o'clock, and on Monday, September 6th, at three o'clock, Mrs. Hewer will lecture on "The Duties of a Monthly Nurse during the Confinement"; on Monday, September 13th, at three o'clock, on "Duties of a Monthly Nurse after Confinement." Admission each time 2s. On Thursday September 9th, and Monday, September 13th, at four o'clock, there will be lectures on midwifery by the medical instructor on "The Mechanism of Labour."

### Everybody's Opinion.

[Correspondence on all subjects is invited, but we cannot in any way be responsible for the opinions expressed by our correspondents. No communication can be entertained if the name and address of the correspondent is not given, or unless one side of the paper only is written on.]

### A QUESTION OF PROPRIETY.

K. H. Monk, Matron, King's College Hospital, writes: We do not employ male nurses in any of our wards. Care is taken on the part of the sisters that no nurses but those well on in their training undertake any delicate matter of nursing in the male wards.

"A Matron" writes: In this hospital all enemata are administered both to male and female patients by the nurses in the ward. In critical cases they are always given by the sister or staff nurse. Probationers are taught to prepare and administer enemata when they have been here about three months. This duty is never allotted to any person other than the nurses in the ward. I may say that we never have any difficulty on this point with male patients. Linen stained at operations is swabbed in cold water for a few hours, then wrung out and sent to the wash in the usual way.

"M. P." writes: Surely "A Medical Correspondent" must know that the administration of enematas are carried out by the staff nurse, not probationer, of a ward. It is usual for the sister to take instructions from the house physician or surgeon, and under her direction the staff nurse acts. In hospitals where there are no sisters the charge nurse administers enematas and all other applications, unless something very simple, which a very new probationer can do; never by other than the nurse are these duties administered. With male patients I never had much difficulty, neither in public nor private. In hospital, when this occurs, I think the best way would be to speak to the house surgeon about the objectionable patient. I am sure he would deal with him as he thinks best. With regard to soiled linen, in most hospitals there is a nurse in charge of the theatre, whose duty it is to collect all soiled towels, &c., that have been used at the operation, which she sends to the laundry as soon as possible. I have answered these questions according to my knowledge of hospital work. One word more. In nursing there should be no question of sex; every patient, whether male or female, is a life put into our hands, and we, as nurses, must do our utmost, and help by kindness and gentleness to lessen the number of objectionable male patients.

### DISTRICT NURSING ON A PROVIDENT BASIS.

Miss Alexander, Heathfield Park, Sussex, writes: I should be much obliged for any information as to whether a system of benefit or other payments in connection with district nursing has ever been successfully worked, either in the country or in towns. Has such a scheme of payments ever been adopted by any association employing only Queen's or fully-trained district nurses for the poor? And, if so, what scale of subscriptions and fees for both general and maternity nursing are found to answer? Information or suggestions on the subject would be a great help to those who are interested in organising district nursing on the wisest basis.

### POINTS IN SICK NURSING.

"Nurse Eleanor" writes: I have read with no small surprise the remarks of Letitia Loyd, in which she seems to think nurses are not quite conscious of the necessity for perfect cleanliness with regard to their patients' cups and glasses or the discomfort caused by a hot, stuffy room. In the first place, it is easy to see she is not a trained nurse, because if so she would know that the great basis of our hospital training is laid on the rules of strictest cleanliness, and I cannot imagine the nurse who would think of having or using anything for her patient which is not fresh and sweet. Secondly, she would seem to fancy that we do not fully realise the importance of maintaining a normal temperature in the room. Surely there are very few nurses so ignorant that they can quite understand the great importance of keeping their patients' room sweet and at a proper heat so as to keep them from danger and at the same time comfortable. I cannot imagine anyone thinking five degrees above or below that required by the doctor of so little importance. As to a nurse sleeping all night and allowing her fire to get low, is more than I can quite take in. However, all I can say is there are careless people in every walk of life, and perhaps "L. L." has been unfortunate enough to come in contact with a careless nurse. But out of justice to myself and fellow-sisters in a beloved calling, I would say preserve me from the criticisms of patients' friends or untrained gossips, who seem to see the faults of others, who are but human, through a jaundiced eye.

### Novelties for Nurses.

### WHOLESOME JAMS.

A large amount of jam goes to relieving the monotony of the nurses' tea table, and the question constantly arises where wholesome jams of moderate price can be procured. Messrs. Pink, Staple Street, Borough, S.E., have turned their attention to supplying this want, and their jams are wholesome, well made, and inexpensive. They also provide other commodities. Their bloater paste is the best we have tasted, and will be a much appreciated addition to the breakfast or tea table. Their lemon squash and sherbet are both very refreshing and of good quality. Their jellies are well flavoured and sold in convenient little packets.

### HOME MADE ICE-CREAMS.

A very clever and simple contrivance has been sent us which places ice-cream within the reach of the smallest household and most limited expenditure. This is an ice-cream freezer introduced by Messrs. E. Roberts, 264, Camberwell Road, S.E. The freezer is a metal cylinder, which has an inner receptacle to contain the mixture to be frozen, whilst the freezing mixture surrounds it. The whole revolves on a stand, the turning being effected by a handle in the easiest manner in from five to ten minutes. Messrs. Roberts sell 3d. packets of mixtures of various flavours, and with the addition of one pint of milk and two to three pennyworth of ice, a pint of delicious ice-cream is procurable for from 6d. to 7d. We have tested the machine, and find that success is gained with the smallest experience. Care should be taken to effectually close the lids of the cylinder and to stir the ice several times during the process of making.

### Appointments.

### MATRONS.

Glasgow.—Miss Ruth Wood has been appointed Lady Superintendent of the Deaconess Nurses' Institution, Glasgow. We are glad to hear that Miss Wood has completely recovered her health. About two years ago she was compelled to resign her post of lady superintendent to the Sick Poor and Private Nursing Association affiliated with Q.V.J.I., through over strain, after having worked with the association for seven years. Her loss was greatly regretted, and the directors gave her a handsome cheque besides a quarter's salary. She also received valuable gifts from her fellow-workers and patients. Miss Wood, before taking up work after her restoration to health, obtained the L.O.S. diploma. She was trained at the Westminster Training School and Home for Nurses, and afterwards became staff nurse, night sister, and out-patient sister of the hospital, and twice acted as temporary matron.

South Shields Workhouse Infirmary.—Miss Anna Mennell was recently appointed Superintendent of Nurses at this infirmary. She was trained for three years at the Workhouse Infirmary, Sunderland, and holds the L.O.S. diploma. She has since worked at the South-Eastern Hospital.

### For Reading to the Sick.

THE HEALING OF ANXIETY.

"Let this mind be in you, which was also in Christ Jesus."

**Verses.**

The while I fain would tread the heavenly way,
Evil is ever with me day by day;
Yet on mine ears the gracious tidings fall,
"Repent, confess, thou shalt be loosed from all."

It is the voice of Jesus that I hear,
His are the hands stretched out to draw me near,
And His the blood that can for all atone,
And set me faultless there before the Throne.

'Twas He Who found me on the deathly wild,
And made me heir of heaven, the Father's child,
And day by day, whereby my soul may live,
Gives me His grace of pardon, and will give.

—*Hymns Ancient and Modern.*

Lord, as to Thy dear Cross we flee,
And plead to be forgiven,
So let Thy life our pattern be,
And form our souls for Heaven.

Kept peaceful in the midst of strife,
Forgiving and forgiven,
O, may we lead the pilgrim's life
And follow Thee to Heaven.

What various hindrances we meet
In coming to the Mercy Seat;
Yet who that knows the worth of prayer
But wishes to be often there.

Have we no words? Ah, think again;
Words flow apace when we complain,
And fill our fellow-creature's ear
With the sad tale of all our care.

Were half the breath thus vainly spent
To Heaven in supplication sent,
Our cheerful song would oftener be,
"Hear what the Lord hath done for me."

**Reading.**

It is not work that kills men, it is worry. We are all agreed upon that. How to avoid worry would be a subject that would enlist a multitude of readers if the recipe could be given. But so much of it comes from causes outside our will, outside our very expectation, that it would be idle to suggest this or that as the way to avoid it altogether. The fact is anxieties will come, and must be met by some all-sufficient remedy. Let us therefore note the reasons which our blessed Lord gives us for their avoidance. "Take no anxiety for the morrow." Why? Because every day, as it brings the returning light of morning, brings also new mercies, new freshness of feeling, new and gracious providences, new opportunities and unexpected deliverances. "The morrow shall take anxiety for the things of itself." And why be so anxious about "things"? The life is more than meat. The training of character, which is immortal, is more than the mere comfort of circumstance which ought to transcend things. You will have enough of these —for the all-sufficient reason which the Master gives, "Your Heavenly Father knoweth what things" ye have need of. Solaces in our great agonies of endurance, which often have no commensurate reason, have therefore no real rest in them for such hearts as ours. From the lip of our Saviour we have such Divine revelations as ought in themselves to furnish perfect rest at all times of anxious wear and worry.—*Rev. W. Statham.*

### Notes and Queries.

**The contents of the Editor's Letter-box have now reached such unwieldy proportions that it has become necessary to establish a hard and fast rule regarding Answers to Correspondents. In future, all questions requiring replies will continue to be answered in this column without any fee. If an answer is required by letter, a fee of half-a-crown must be enclosed with the note containing the enquiry. We are always pleased to help our numerous correspondents to the fullest extent, and we can trust them to sympathise in the overwhelming amount of writing which makes the new rules a necessity. Every communication must be accompanied by the writer's name and address, otherwise it will receive no attention.**

*Superfluous Hairs.*

(365) 1. Can anyone tell me the name of a medical man who removes superfluous hairs from the face by electricity? 2. Is this the only permanent cure?—*Nurse.*

1. Write to the toilet department of a ladies' journal. 2. We believe so.

*A Book on Nursing.*

(366) 1. I should like to get a book on nursing that goes into the causes as well as the effect of treatment of diseases. 2. I should be obliged if you can tell me of a medical dictionary giving more information than Hoblyn but simpler than Quain.—*Crouch End.*

1. "Nursing: its Theory and Practice," a text-book for nurses, by Percy G. Lewis, M.D., may suit you. The price is 3s. 6d. It is published by the Scientific Press, 28 & 29, Southampton Street, Strand. "Nursing," by Isabel Adams Hampton, price 7s. 6d. net, from same publishers, is widely used. 2. "A Dictionary of Medical Science," price 30s. (21st edition), Richard J. Dunglison, A.M., M.D.; publishers, J. and A. Churchill, 7, Great Marlborough Street.

*Midwifery Fees.*

(367) I am a certificated monthly nurse, and was engaged to nurse a lady in August, but her baby came in June, and now I am left without a case. I and my solicitor have written to the lady asking for part of my fees, but have received no reply. Can I County Court the lady, or am I likely to get my fees?—*Hospital Nurse.*

You are entitled to half fees, and will recover the debt through the County Court.

*Red Cross Nurses' Association.*

(368) Would you kindly let me know if there is a branch of the Red Cross Nurses' Association in Manchester, and where it is?—*M. J. F.*

There are no branches in England.

*An Aged Nurse.*

(369) Can you or any of your readers tell me of any home or fund for aged nurses? I know a most deserving case of a nurse between fifty and sixty years of age whose failing sight incapacitated her for work. The little money she had saved has been spent during the last two years. She has undergone two operations for her eyes and fears that a third will soon be necessary.—*B. C.*

Apply to Secretary, Trained Nurses' Annuity Fund, 72, Cheapside, E.C. B. C. would also be eligible for a general charity. See "Englishwoman's Year Book" for list.

*The Shape of a Pad.*

(370) Will you kindly tell me what kind of pad should be made for a misplaced kidney and how it should be worn. I am wearing an abdominal belt and have been told to make a pad also.—*One in Pain.*

Ask a nurse to show you, or call at your nearest nursing association and you will see one.

*Subjects Required for Chemist's Examination.*

(371) 1. Will you kindly inform me what subjects are necessary to qualify for a chemist's examination? 2. What premium I should have to pay to walk a hospital such as St. Mary's, Paddington, or St. George's, Piccadilly?—*J. Millwaters.*

1. Write to the Secretary, Pharmaceutical Society, Bloomsbury Square, W.C. (2) And to the secretaries of these hospitals, who will give all information.

*Postage for "The Hospital."*

(372) Will you kindly say whether I can send The Hospital, with "Mirror" Supplement, by post for a halfpenny weekly? I have been doing so for the last four months, and the nurse to whom I sent it has had to pay an extra penny when she received it.—*Nurse.*

The Hospital and "Mirror" are registered as one newspaper, so the postage is one halfpenny. Will you kindly send names of post offices from and to which it was sent, as the manager will make inquiries.

*Medical and Surgical Nursing.*

(373) 1. I should feel obliged if you could give me the name of a good book on medical and surgical nursing, a book giving the causes, effects, and treatment, if possible. 2. I should prefer an early answer by post, though I take your newspaper.—*Miss Sevenoaks.*

1. See answer to (366). Those named might help you, but you want an impossibility. Can one book contain the arts of medicine, surgery, and nursing? and, if so, would it be safe in other than the physician's or surgeon's hands? 2. An answer by post costs 2s. 6d.

*A Visiting Monthly Nurse.*

(374) Do you know of any institution, or any nurses in the neighbourhood of Kensington where a monthly nurse could be obtained for one or two hours daily?—*Sister Annie.*

There are several homes in this neighbourhood from which a nurse might be engaged. A list will be found in the Post Office Directory. The Lady Superintendent, Holy Cross Society of Trained Nurses, 38, Ladbrook Road, W., would probably know some one. The society is Roman Catholic. The lady's medical man might be able to recommend a nurse. Miss Wood, The Nurses' Hostel Co., Ltd., Francis Street, W.C., would also provide a nurse on these terms.

